# Reproducibility of Water Diuresis: Implications for Establishing the Beverage Hydration Index of a Sugar-Free Caffeinated Electrolyte Beverage

**DOI:** 10.3390/nu18193279

**Published:** 2026-10-06

**Authors:** Scott D. Bassett, Brian Hack, Alec Harp, Melinda Millard-Stafford

**Affiliations:** 1 Exercise Physiology Laboratory, Georgia Institute of Technology, Atlanta, GA 30332, USA; scott.bassett@gcsu.edu (S.D.B.); bhack@gatech.edu (B.H.); aharp6@gatech.edu (A.H.); 2 School of Health & Human Performance, Georgia College & State University, Milledgeville, GA 31061, USA

**Keywords:** sodium, potassium, fluid balance, caffeine, sex, diuresis

## Abstract

Background/Objectives: The Beverage Hydration Index (BHI) compares fluid retention of beverages relative to water based upon urine output over time. We examined the reliability of diuresis from water ingestion and its potential impact on the BHI of a sugar-free energy drink with electrolytes (CAF). Methods: Twenty-four adults completed two trials consuming either one liter of water or CAF over 30 min with urine output measured over 4 h. In a sub-group (*n* = 15), water trials were repeated twice (~6 months apart) to compare the impact on BHI of CAF. Results: Urine mass was not different (*p* = 0.46) between repeated water trials; however, intraclass correlations indicated poor reliability (r = 0.11, r = 0.31) at 2 and 4 h. Mean (±SD) BHI of CAF based upon two water trials did not differ (*p* = 0.44) at 2 h (0.90 ± 0.17 versus 0.97 ± 0.22) and 4 h (0.99 ± 0.18 versus 1.01 ± 0.21). The BHI for CAF at 60 min (0.75 ± 0.21; 0.83 ± 0.23) was the only time point lower (*p* < 0.001, *p* = 0.008, respectively) than a BHI of 1.0 (water control). The BHI of CAF for all participants had similar results: no difference between CAF and water from 2 to 4 h. Conclusions: Although the mean diuretic action of water ingestion appeared similar across repeated trials, the reproducibility of diuresis within individuals was poor. Despite low reliability, the impact on establishing the BHI of CAF was not significant. CAF provided similar hydration benefits as water from 2 to 4 h (varying by 1–7%) despite increased diuresis only in the first hour, suggesting that a sugar-free electrolyte beverage containing caffeine supports long-lasting hydration. Moreover, the establishment of BHI metrics is robust to interference from potential differences in individuals’ pre-test free-living conditions.

## 1. Introduction

General guidelines for achieving an adequate daily total water intake have been defined for various populations around the globe [1,2]. Adequate total water intake varies from a minimum of 1 L per day [3] to 2 L in women [2] or 3 L for men [1], depending upon environmental exposures and levels of physical activity of the individual. Most total water intake is derived from drinking beverages [4], defined as any potable liquid intended for human consumption. In addition to water, beverage categories include juices, tea, coffee, soft drinks, and alcohol. While all beverages supply fluid, the question becomes whether the hydration potential differs among these beverages when compared to plain drinking water.

The hydration process involves several steps. First, fluid must be palatable for oral consumption, then absorbed from the gastrointestinal tract and finally either retained or excreted by the kidneys depending on hydration status and other hormonal influences. The Beverage Hydration Index, or BHI, was developed by Maughan et al. in 2016 [5] to assess the relative fluid retention of a beverage compared to water as a practical model to scale the hydration potential of beverages along a continuum. BHI is a ratio derived from the amount of urine produced over several hours after drinking one liter of water divided by urine produced from drinking one liter of an experimental beverage. If urine output is greater for water, this results in a BHI ratio above 1.0, indicating the beverage has greater fluid retention relative to water. However, if the BHI ratio is less than 1.0, the beverage results in greater diuresis, or more urine output relative to water.

The BHI model has since been utilized to compare the hydration properties of a variety of beverages [6,7,8,9,10,11,12] and studies have demonstrated that beverage composition can influence the BHI. The original investigation [5] indicated milk, orange juice and oral rehydration solutions had a BHI ratio significantly greater than that of water (or 1.0). Subsequent studies also validated the addition of electrolytes to water increased the BHI [8,10] compared to plain water. Increasing the sugar content improved the BHI, unlike that observed with systematically increasing the level of caffeine (up to 400 mg per liter) [12], which had no effect on BHI. This was somewhat surprising given the common messaging and meta-analysis reporting that caffeine acts as a mild diuretic when consumed in resting conditions, although this small effect is negated during exercise [13]. However, caffeine habituation status may attenuate caffeine’s diuretic effect at rest [14,15].

Caffeine continues to be of particular interest regarding the impact on hydration status. The original BHI study [5] indicated that common caffeinated beverages (e.g., tea, coffee, diet soda) had lower BHIs versus water, although deemed not significantly less hydrating compared to water. New caffeinated products such as energy drinks have now appeared, with no common definition for ingredients. Millard-Stafford et al. [11] compared a variety of commercially available caffeinated energy drinks containing different amounts of sugar (from 0 to 24 g/L) and varying levels of electrolytes (0.6–45 mmol sodium) when consumed with equal parts (500 mL each) water. Energy drinks with sugar and higher sodium tended to elicit higher BHIs compared to others, but due to the differential levels of caffeine, conclusions about the optimal energy drink formulation that simultaneously provides optimal hydration remain challenging.

Factors such as body mass and sex appear to have minimal impact on the reproducibility of the BHI [16]. However, sex did appear to influence a greater fluid retention of caffeinated energy drinks in women compared to men, despite consuming greater relative amounts of fluid and caffeine relative to body mass when using the absolute fluid dose administration of the BHI protocol [11]. Although attempts to control pre-test dietary intake are usually undertaken, it is possible that pre-test nutrition (e.g., sodium, protein and water) might impact the “baseline” diuresis of the water control trial. Seasonal variation in environmental exposures might also influence the repeatability of the measurements of fluid retention used in the model. Because water serves as the reference condition, poor reproducibility of the diuretic response could affect the interpretation of any test beverage of interest. Nonetheless, the BHI is considered a valid model to assess short-term beverage hydration characteristics under well-controlled conditions when individuals remain resting and begin in a euhydrated state.

Our purpose was to determine the reproducibility of the diuretic action of repeated water trials for men and women. If reliability is poor, this could have downstream effects on the calculation of the BHI with another beverage of interest. Sollanek et al. [16] determined that the variation in urine output for seven participants (four women) after drinking 1 L of water during three separate trials was ±130 mL (11% CV), and Maughan et al. [5] originally reported drink repeatability of ±180 mL for 12 participants, although the drink was unspecified. Whether potential differences in water ingestion (the numerator of BHI) are meaningful in calculating BHI when the denominator is a novel test beverage remains unclear. Thus, we chose to evaluate the impact of repeated water ingestion trials on assessing the hydration potential of a sugar-free, caffeinated electrolyte beverage based on the BHI calculation from two different water comparisons separated by season. We hypothesized that urine output after repeated water ingestion would be reproducible within individuals and would, therefore, not meaningfully alter the calculated BHI for a test beverage. We further hypothesized that the test variable, a sugar-free, caffeinated electrolyte beverage, would not significantly impair fluid retention compared to water over the BHI assessment period.

## 2. Materials and Methods

### 2.1. Study Population

Twenty-four adult men (*n* = 14) and women (*n* = 10) were recruited to participate in the study. All gave their written informed consent as required by the University Institutional Review Board (Protocol H23495) in accordance with the guidelines in Declaration of Helsinki. All were healthy young adults not classified as obese (<30% and <35% body fat for men and women, respectively). Participants were restricted from taking over-the-counter drugs or medications influencing fluid balance in the days preceding their experimental visits. Women were not restricted from participation based on the use of oral contraceptives, and menstrual cycle phase was not systematically controlled during scheduled visits. Out of the 10 total women, only one was taking oral contraceptives. A sub-group of 15 participants (10 men, 5 women), performed an additional water ingestion trial approximately 6 months apart to establish reliability of the diuretic action of water ingestion. No women out of five in this sub-group for reliability were taking oral contraceptives.

Habitual intake of caffeine was not an inclusion criterion due to our prior study indicating no differential effect on the BHI for a caffeinated beverage [11]. Caffeine naïve was defined as <25 mg/day [17] based on a 7-day caffeinated food/beverage frequency questionnaire [17] converted to mg of caffeine based on published sources [18,19]. For the full sample (*n* = 24) to evaluate the BHI of CAF, 10 were naïve and 14 were habitual caffeine users. For the reliability study, ten participants (five female) were classified as habitual users and 5 participants (male) were considered naïve. Estimated caffeine intake ranged from 0 to 370 mg per day with a mean caffeine intake of 136.6 ± 77.7 mg/day. No participant exceeded a daily caffeine level (400 mg/d) generally considered to be high or unsafe [20].

During a familiarization session, height, weight and body composition were assessed using the seven-site skinfold method for men and women [21,22]. Total body water was estimated using a multi-frequency bioimpedance instrument (InBody 770, Seoul, South Korea). Total body water was also estimated [23] based on fat-free mass obtained with skinfolds. Table 1 indicates the mean (±SD) physical characteristics and habitual caffeine intake of participants who completed all trials.

### 2.2. Study Design

Experiments were performed in the Exercise Physiology Laboratory at the Georgia Institute of Technology, Atlanta, GA. This study was a crossover, quasi-experimental study using a within-subjects, repeated measures design (i.e., each participant completed all beverage trials). Double blinding was not possible due to the use of tap water in the repeated water trials. However, participants were unaware of the experimental beverage being tested. The water ingestion trials were repeated twice (one winter and another in late spring/summer ~6 months apart). Only the order of the winter water trial and experimental caffeine beverage trial were counterbalanced and performed in the same season, typically within one to two weeks. All trials began between 0700 and 0900 h with time of day for testing held constant for each subject. Each test session was conducted in thermoneutral conditions (21 °C). Participants refrained from strenuous activity for the 24 h before each test day and recorded 24 h dietary intake (including beverages). Participants were instructed to replicate a similar dietary intake on subsequent visits and a copy of their food records were provided to them for this purpose.

### 2.3. Test Beverages

The contents of the test beverage were blinded to participants but not the water control trials. The test beverage was a patent-pending sugar-free, caffeinated electrolyte beverage containing allulose, and a proprietary amino acid blend (L-glutamine, L-alanine) and stevia leaf extract to impart sweetness without raising blood glucose. It was not a commercially available product at the time of the experiment. The test beverage was reconstituted with 950 mL tap water added to 28 g of powder from sealed packets. One liter of this reconstituted caffeinated-electrolyte beverage (CAF) was served and contained 210 mg caffeine, 115 mg sodium [~44 mmol], and 882 mg [~18 mmol] potassium. This electrolyte blend is considerably higher than a traditional sports drink but contained no sugar. The test beverage osmolality was 225 mosmol/kg and did not have a similar coloring to water (pale yellow). Beverages were mixed fresh, stored overnight in a refrigerator and served from a sealed container (at ~10–12 °C) but sat at room temperature for the 30 min ingestion period. The beverage administration resulted in lower relative fluid volume (*p* < 0.001) for males (13.4 ± 1.4 mL/kg body mass) versus females (16.9 ± 2.0 mL/kg body mass) and lower caffeine dose (2.8 ± 0.3 vs. 3.6 ± 0.4 mg/kg body mass) for males.

### 2.4. Test Protocol

All experimental sessions were completed in a temperate (~21 °C) room. Participants were asked to replicate their food and fluid intake based on a diet log (results in Table 2) and avoid exercise 24 h prior to each test session. Differences in nutrient intake between the two water trials were not significant, but water trial 2 tended to have higher protein intake (mean difference of 26 gm, *p* = 0.13) compared to water trial 1. Otherwise, the mean differences for sodium or water intake were relatively small across all three trials.

To establish euhydration prior to the experimental sessions, participants were instructed to ingest an extra liter of water (~1800 and 2300 h) in addition to their normal daily intake and fast (both food and fluid) ~8 h until their morning test. Tap water was provided (500 mL) to consume within 60 min prior to arriving for the laboratory experiment and timing of this ingestion was repeated prior to each trial. Participants emptied their bladder upon waking, retaining a mid-stream sample in a sterile collection cup. Urine specific gravity was measured by refractometry (Atago, URC-Ne Refractometer, Tokyo; Japan) and found to be similar across the three beverage trials (1.015 ± 0.005, 1.017 ± 0.004 and 1.016 ± 0.005). If this first-catch urine specific gravity was >1.025, indicative of possible hypohydration [24], the participant was re-scheduled.

**Figure 1:**
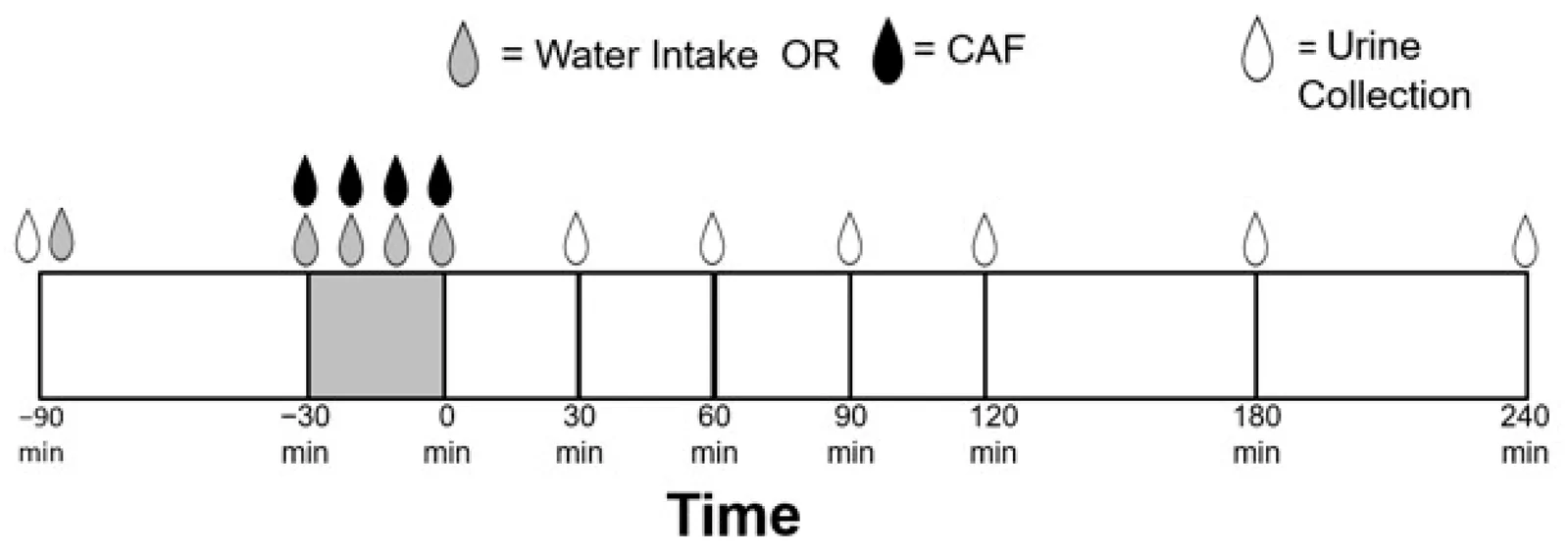
Schematic of the Beverage Hydration Index test protocol performed for up to two water trials and one caffeinated beverage (CAF) trial.

A schematic of the BHI test protocol is depicted in Figure 1, based on the original protocol cited in the literature [5]. Participants were given one liter of either water or the test beverage over 30 min (four equal aliquots ingested every 7.5 min). After ingesting the beverage, participants remained seated, measured their body mass and completed a beverage rating form. The post-ingestion observation period was 240 min in duration after drinking the one-liter bolus during which urine mass, body mass was obtained. This protocol timeline was similar to previous studies [10,11]. No food was provided for the entire test session.

Body mass and urine mass were measured on a precision digital platform scale (Ohaus Defender 5000 platform scale, Ohaus Corporation, Parsippany, NJ, USA). Male participants collected urine directly into a 3 L plastic 24 h urine container (Parter Medical Products, Carson, CA, USA), while the women utilized Vakly Graduated Specimen Collector Pans (Avaline Medical LLC, Lakewood, NJ, USA) placed under the toilet seat. Urine cumulative mass at each time point was measured to the nearest 1 g, with the mass of the empty container tared to subtract from total urine mass. BHI was calculated as the ratio of cumulative urine mass for tap water divided by the cumulative urine mass of the test beverage at each time point as originally described [5]. Urine sodium and potassium were measured with Horiba LAQUA twin (Horiba, Kyoto, Japan) compact ion meters. Cumulative sodium and potassium loss was calculated based on the sum of urine mass multiplied by electrolyte concentration at each hourly time point.

### 2.5. Statistical Analysis

A two-factor analysis of variance with repeated measures (beverages × 4 time points) was used for drink comparison measures except BHI. Since water is designated as the control (constant BHI score of 1.0 with no SD among participants), the BHI comparison requires a non-parametric rank-based Friedman’s test [25] and use of a one-sample *t*-test to compare test beverage BHI against the 1.0 value for water. Water trial 1 and 2 comparisons (*n* = 15) at 120 and 240 min time points were performed with paired *t*-tests and an intra-class correlation coefficient (ICC). A mixed ANOVA (4 time points as the within subjects repeated measure) with a between-group factor (sex) was used to compare male versus female CAF BHI. Data were analyzed using SPSS version 29 (IBM SPSS Statistics for Windows, Version 26.0. Armonk, NY, USA: IBM Corp.). Post hoc multiple comparisons were corrected using the Bonferroni method. An alpha level of *p* < 0.05 was chosen to denote statistical significance. Data are presented as mean (±SD) unless indicated otherwise.

The original BHI study across multiple test sites used 24 participants [5] per drink trial, and in subsequent studies [6,7] sample sizes of 10–12 participants were verified to have sufficient statistical power. Our effect size calculations were based on BHI data reported previously [5,6,7]. The power analysis was conducted using G*Power 3.1.9.4 for a within factors repeated-measures ANOVA examining the main effect of beverage on BHI. Assuming Cohen’s f = 0.35, α = 0.05, power = 0.80, and correlation of repeated measures r = 0.50, a sample size of 14 participants was determined to be sufficient to detect differences among two beverage conditions (across 4 time points). Since a previous report [16] indicated no difference in BHI based on either sex or body mass, both men and women were combined and treated as one group. Therefore, fifteen participants completed all trials but with unequal numbers of men and women, meeting the sample size needed to detect differences for the water repeatability trials.

## 3. Results

### 3.1. Urine Output: Reliability of Water Trials (n = 15)

**Figure 2:**
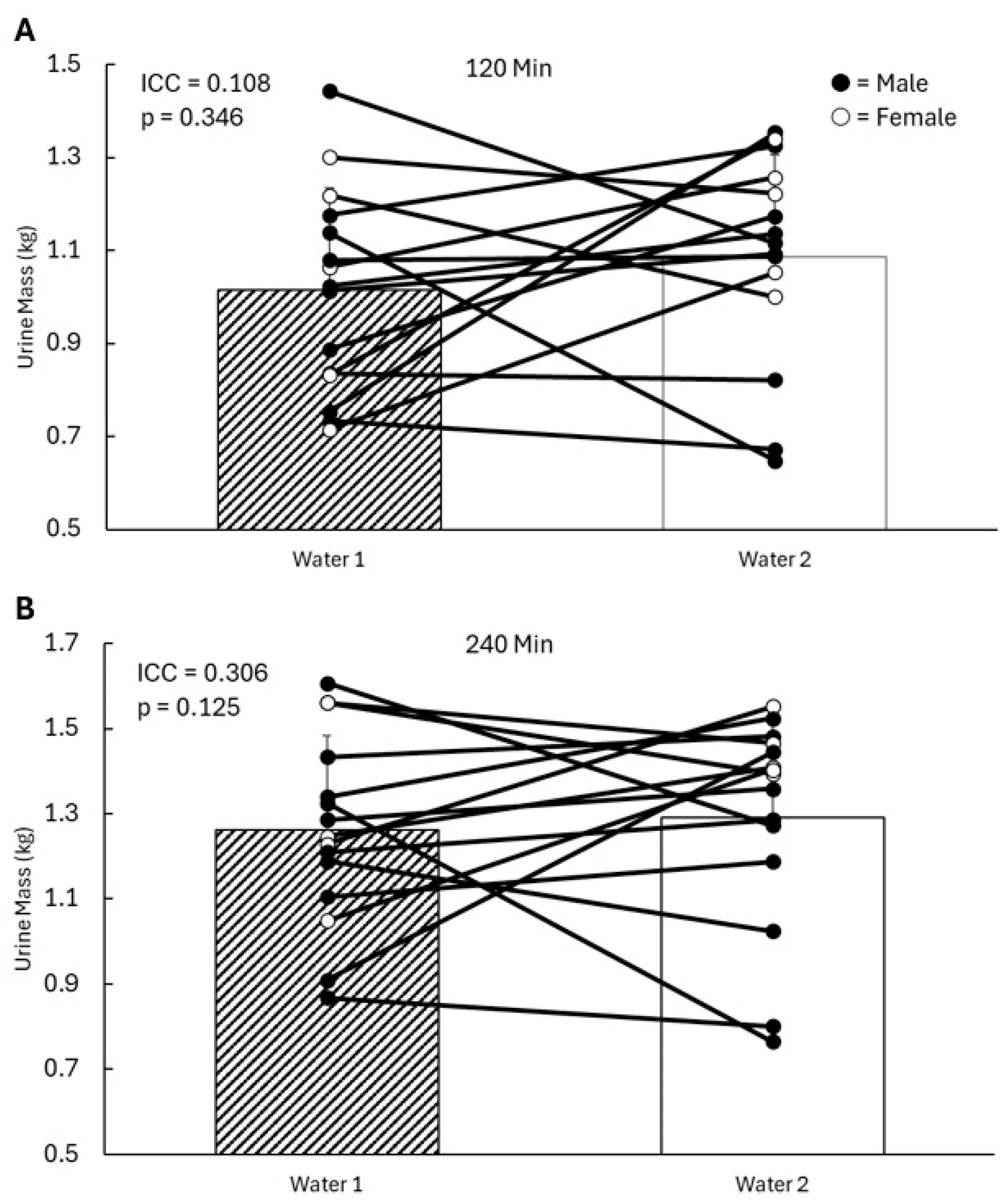
Mean ± SD urine mass in repeated water trials (*n* = 15) at 120 (**A**) and 240 min (**B**). Individual participant values are depicted with solid lines and exhibited poor intra-class correlations (ICCs) which were not significant at 120 or 240 min (*p* = 0.35, *p* = 0.125, respectively). Male participants in closed circles, females in open circles.

Urine mass was not different (*p* = 0.46) across all time points between repeated water trials. Figure 2 illustrates the mean and individual urine mass values for repeated water ingestion trials 1 and 2 at 120 (top) and 240 min (bottom) post-ingestion. Based on paired *t*-tests, differences in mean urine mass after water ingestion (72 and 30 g at 120 min and 240 min, respectively) were not significantly different (*p* = 0.36, and *p* = 0.68), with small (0.2) and trivial (0.1) effect sizes. However, the ICCs to evaluate reliability were poor at both 120 and 240 min and not significant, as shown in Figure 2. Individual responses (identified by sex) are plotted with inconsistent results (6 out of 15 participants were higher on water 1). Sensitivity analysis omitting three divergent responders revealed an improved ICC = 0.54 (*p* = 0.03) and 0.63 (*p* = 0.01) at 120 and 240 min.

### 3.2. Urine Mass and Cumulative Electrolyte Loss (n = 15)

**Figure 3:**
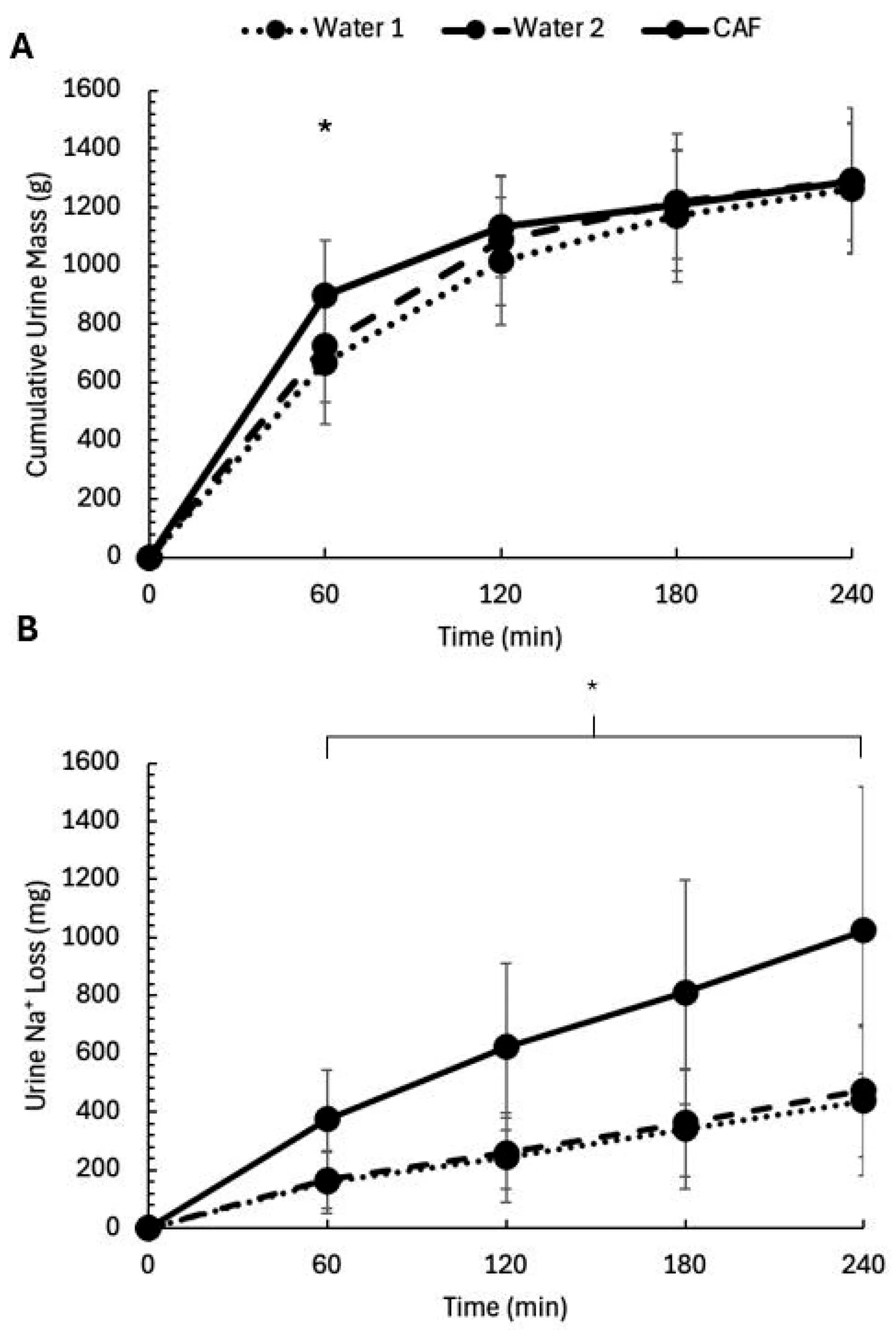
Panel (**A**): Mean (±SD) cumulative urine mass for water repeated trials and CAF (*n* = 15). * Greater diuresis at 60 min in CAF vs. Water 1 and 2 trials, *p* < 0.001, *p* = 0.019. Panel (**B**): Urine Na^+^ loss higher (*p* < 0.001) in CAF from 60 min to 240 min.

Cumulative urine mass is plotted in Figure 3A over 240 min. There was no main effect among the three drinks (*p* = 0.23), but a significant drink × time interaction (*p* < 0.001). CAF was higher but only at 60 min compared to water 1 (*p* < 0.001) and water 2 (*p* = 0.019). By 240 min, the mean difference was only 30 g between water trials and CAF within 26 and 4 g of water 1 and 2, respectively. Cumulative Na^+^ loss over time is plotted in Figure 3B. There were significant main drink effects, time effects, and drink x time interaction effects (all *p* < 0.001). Urinary Na^+^ loss was greater (*p* < 0.001) with CAF from 60 to 240 min compared to both water ingestion trials which were not different (*p* = 1.0) from each other at any time point. By the end of 240 min, urinary loss of Na^+^ was 587 and 551 mg greater with CAF compared to water 1 and 2 ingestion trials. The same relationship was found with cumulative K^+^ loss over time with significant drink, time, and drink x time interaction effects (all *p* < 0.001). By the end of 240 min, urinary loss of K^+^ was 529 and 536 mg greater for CAF compared to water 1 and 2.

### 3.3. Beverage Hydration Index

**Figure 4:**
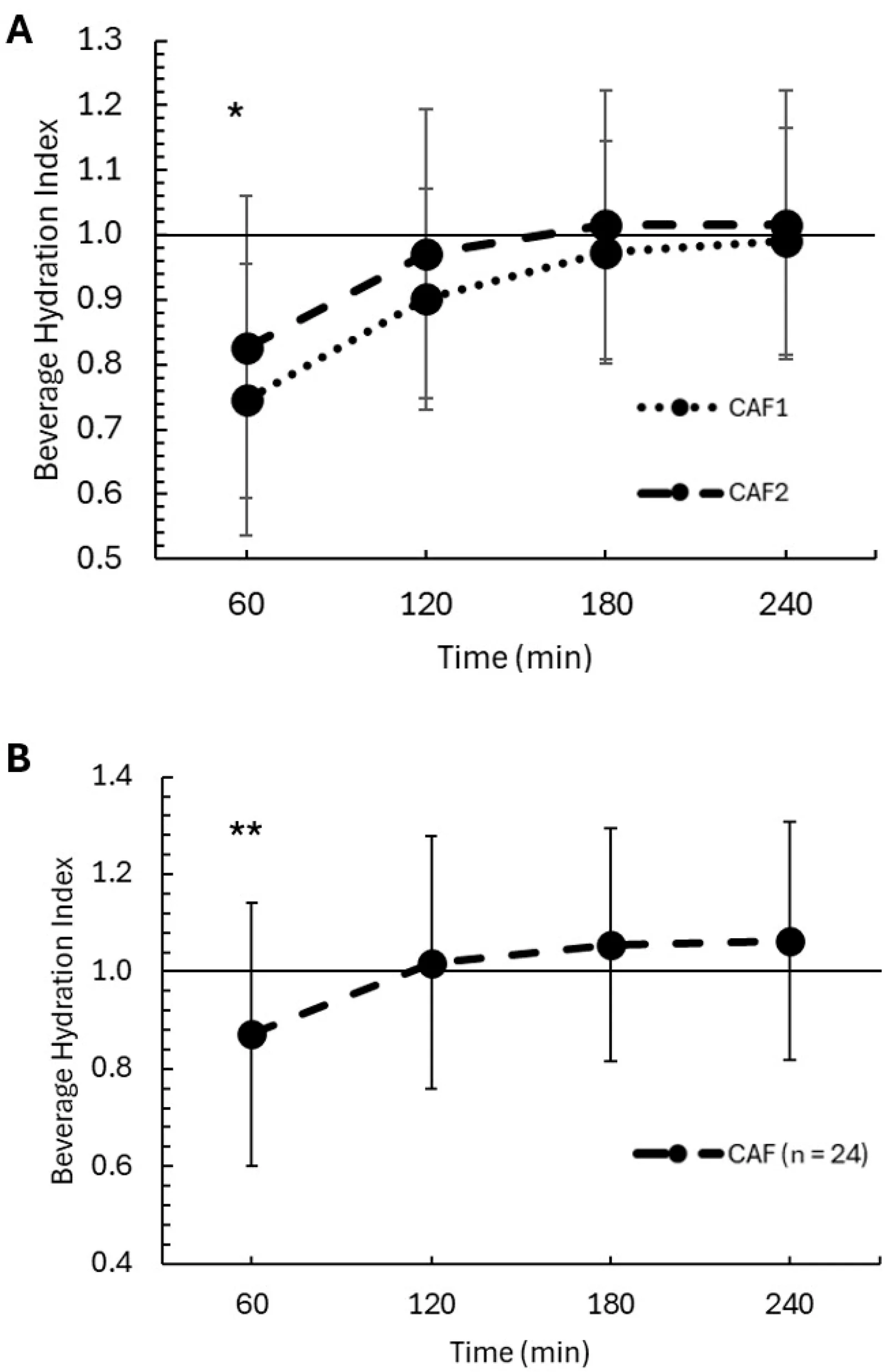
(**A**) Mean (±SD) Beverage Hydration Index for sugar-free caffeinated electrolyte beverage (CAF) based on two water control trials. * BHI for CAF1 and CAF2 (based on Water trials 1 and 2) lower than 1.0 (*p* = 0.001, *p* = 0.008) indicated by the solid reference line across at 1.0 BHI. (**B**) Mean (±SD) Beverage Hydration Index for CAF based on water control trial 2 (*n* = 24). ** CAF at 60 min is lower than 1.0 and also compared to all subsequent time points (120–240 min).

In Figure 4A, BHI for CAF is plotted over time based on water 1 (CAF1) and water 2 (CAF2). CAF elicited a significantly lower BHI (*p* = 0.001; *p* = 0.008) compared to the water standard of 1.0, but this was only observed at 60 min. The BHI mean for caffeine < 1.0 (0.83 and 0.75) in the first hour was due to approximately 17–25% lower fluid retention compared to water, with CAF eliciting a greater diuresis by 231 ± 178 g and 174 ± 209 g (*p* < 0.001, *p* = 0.019), respectively. But these BHI values for CAF were not significantly different (*p* = 0.29) from each other at 60 min based on the water trial utilized. However, over time, the difference in BHI calculated for CAF was only 7% and 1%, by 2 and 4 h, respectively. None of these time points (120 through 240 min) differed from the water control value of 1.0. An average BHI for CAF across all time points (60–240 min) was 0.90 and 0.96 based on water 1 and water 2 trials.

Figure 4B plots BHI with all 24 participants (additional 5 women, 4 men combined with the reliability sub-group of 15), based on water control trial 2, to compare with CAF. Similar to the sub-group findings, BHI for CAF (0.87 ± 0.27) was only lower (*p* = 0.022) than water control (1.0) at 60 min. Thereafter, up through 240 min the BHI for CAF was not different from 1.0 (with mean values slightly above 1.0 ranging between 1.02 and 1.06). There was a drink × time interaction effect (*p* < 0.001) over the 240 min with *n* = 24, but not with *n* = 15 in Figure 4A (*p* = 0.08). In addition to being lower than water (1.0) at 60 min, the CAF BHI at 60 min was lower (*p* = 0.029) compared to 120–240 min.

**Figure 5:**
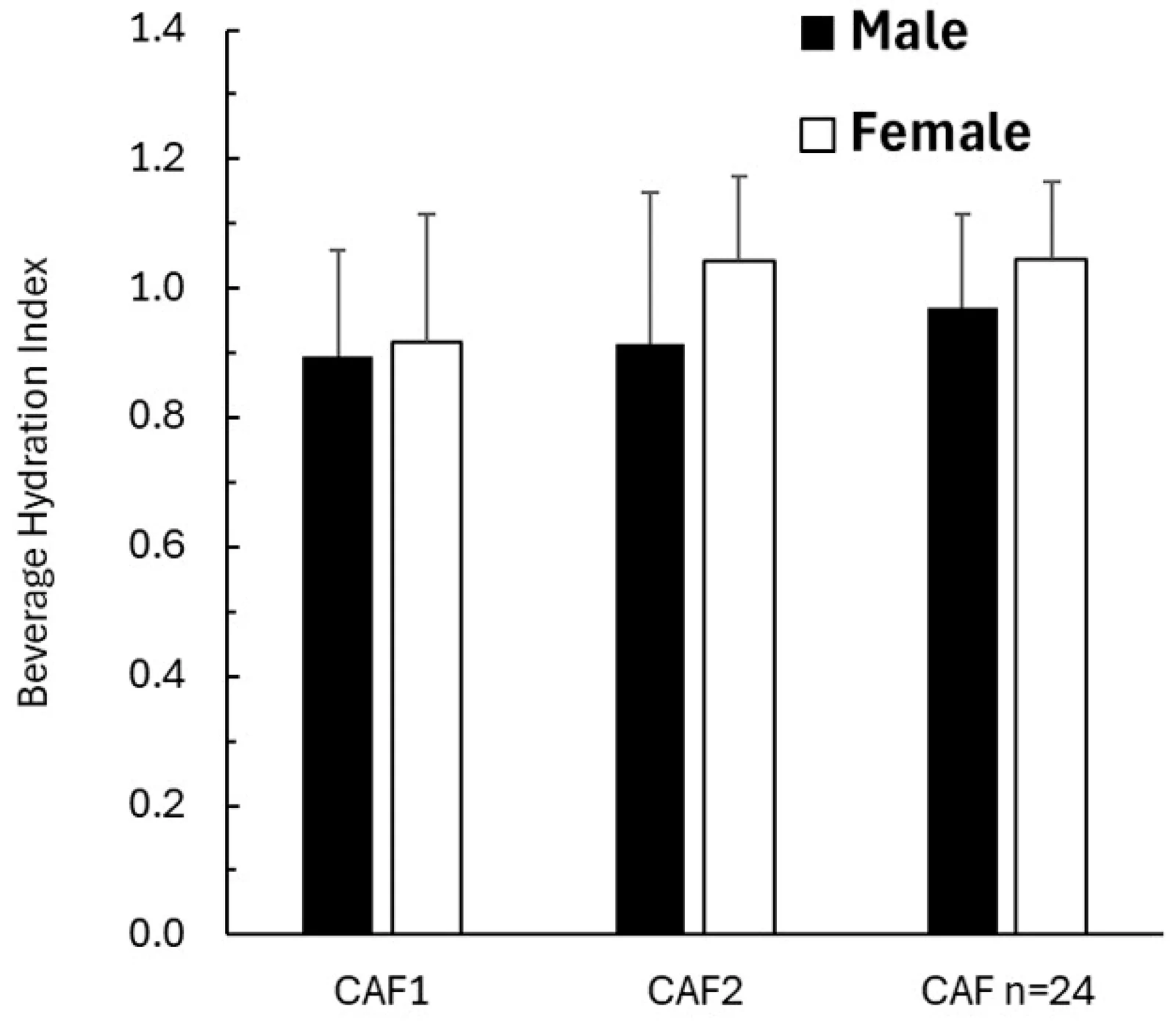
Mean (SD) BHI for sugar-free, caffeinated electrolyte beverage (CAF) based on all time points (60–240 min), averaged for water trial 1 (CAF1) and water trial 2 (CAF2), grouped by male (*n* = 10) and female (*n* = 5) participants. CAF *n* = 24 represents mean BHI averaged across all time points based on all 24 participants (10 female, 14 male). No BHI difference between groups for any of the comparisons by sex.

The BHI for CAF averaged across all time points (60–240 min) during water trials 1 and 2 were compared for male and female participants (Figure 5). There was no difference by sex (*p* = 0.82; *p* = 0.29) for CAF BHI based on water 1 and 2 trials, respectively. CAF BHI was 0.90 ± 0.16 and 0.92 ± 0.23 for men, 0.92 ± 0.20 and 1.04 ± 0.13 for women, respectively. Mean CAF BHIs for both groups tended to be slightly higher based on water 2 (the winter trial scheduled in closer time proximity to CAF) but water 1 and 2 did not differ within males (*p* = 0.82) or females (*p* = 0.33). The CAF BHI for all participants (*n* = 24) based on water trial 2 (averaged across each hourly time point) is also presented in Figure 5 (right bars). The mean CAF BHI for female participants (1.04 ± 0.25) was not different (*p* = 0.47) compared to males (0.97 ± 0.22).

## 4. Discussion

Our hypothesis that the diuretic action observed during two water control ingestion trials would be reproducible and not impact the derivation of BHI for a sugar-free, caffeinated electrolyte beverage was only partially supported. In contrast to our hypothesis, the reliability of urine mass output at key time points (120 and 240 min) post-ingestion of one liter of water was poor. However, despite significant individual variability in the diuretic action following controlled water ingestion in a few individuals, the resultant calculated BHI was only marginally affected and, therefore, robust as hypothesized. Moreover, the test beverage, a caffeinated electrolyte drink containing no sugar, demonstrated similar hydration benefits compared to water over four hours. Despite a short-term diuretic effect in the first hour, overall fluid retention and the BHI of the caffeinated beverage was no different than water across all other time points, and similarly effective in both men and women.

### 4.1. Reproducibility of Water Diuresis

Water excretion via the kidneys in response to drinking one liter of water was not reproducible in the present group of participants. The fact that some individuals (and not exclusively the women per se) had large differences in water retention at both 120 and 240 min post ingestion, as shown in Figure 2, was unexpected and challenging to explain. Sollanek et al. [16] determined the mean variation in urine output after 1 L water ingestion was ±130 mL (11% CV) while Maughan et al. [5] reported ~±180 mL (18%CV). The latter study reported, 4 h after drinking 1 L of water, that urine mass across three different test sites was similar among each group of 24 participants (~1.3 L). Our mean difference in urine mass was also relatively small, only 72 g (~7%) between water trials 1 and 2, but the SD was 295 g at 120 min (and a mean of 30 ± 277 g at 240 min). At 240 min, the mean urine mass loss was also ~1.3 L across all trials, similar to the original BHI paper [5]. In nine individuals, urine mass varied by <150 g at 4 h across the two trials. Notably, three individuals had the most divergent responses (2 men, 1 woman). One male participant reported with a similar first-catch urine specific gravity (1.015, 1.013) but lower urine mass (by 500 and 560 g) at 2 and 4 h on water trial 2. Another male participant who reported with consistent but somewhat higher urine specific gravity (1.024 and 1.023) had higher urine mass (by 600 and 535 g) on water trial 2. One of the five women with a first-catch urine specific gravity of 1.011 and 1.014 had higher urine mass (by 508 and 323 g) at 2 and 4 h on water trial 2 and was not taking oral contraceptives. Despite the poor ICC for water repeatability (*n* of 15) shown here, the SE is ~74 g (based on SD of the difference using 286 g (averaging the SD across 2, 4 h)/√15) with 95% CI = ±145 g which is within the reported range of ~130–180 g reported by Sollanek et al. [16] and Maughan et al. [5], respectively. This study further illustrates the key point that variability in some individuals’ diuretic response to drinking water may not have a meaningful impact when the denominator determining a mean BHI is another test beverage that is being compared.

### 4.2. Biological Sex Differences

The impact of sex on the BHI has been addressed previously by Sollanek and colleagues [16], stating that neither BMI nor sex would impact this metric. Hormonal fluctuations throughout the menstrual cycle may elicit small changes in body mass (gain of ~0.5 kg during menstruation), possibly related to extracellular fluid retention estimated using single-frequency bioelectrical impedance [26]. However, renal excretion (electrolytes, water) was not affected [27] in a carefully controlled study manipulating estrogen and progesterone post-ovulation, although progesterone-related increases in aldosterone were reported. Menstrual cycle phase did not have independent or additive effects on body mass loss or urinary indices, including 24 h urine volume [28]. When observing sixty women over one year of menstrual cycles, self-reported fluid retention scores were not different between ovulatory and anovulatory cycles, and neither estradiol nor progesterone were associated with fluid retention scores [29]. Nearly all of the women that were part of our study were not taking oral contraceptives. The literature does not support differences in fluid retention or electrolyte balance for women during post-exercise rehydration during different phases of the menstrual cycle [30], although the means may obscure potential individual differences and more investigation is recommended [31]. Consequently, the poor reliability in the individuals’ diuretic response elicited by water ingestion in the present study does not appear to be fully explained by sex differences or the menstrual cycle per se.

### 4.3. Nutritional Factors

Dietary factors are believed to influence diuresis and, therefore, may influence the BHI metric if not adequately controlled during pre-test conditions. Some of the more important factors have recently been reviewed [32]. To that end, our participants were instructed to replicate their food and beverage intake for 24 h prior to each experimental trial to minimize fluctuations in potential confounding effects.

High dietary sodium has typically been cited to be associated with increased urine production in human studies [33]. Moreover, our instructions to participants emphasized maintaining a lower sodium intake along with providing a list of high-sodium foods to avoid. Therefore, the mean difference in sodium between the two water trials was modest (~250 mg). One male individual cited above with lower urine mass in water 2 (winter) and similar pre-test urinary specific gravity recorded a 60% lower sodium intake (by 2.4 g) and 15% lower water intake (by 650 mL). The other two participants with a large discrepancy in water diuresis had very little difference in sodium intakes (within 250 mg). In hypertensive individuals, urine volume was reduced when a very high salt intake (10 g/d) was reduced to very low intake 1 g/d [33]. In contrast, other clinical human studies reported increased salt intake from 0.6 g/d to 24 g/d did not change fluid intake or urine volume [34], and increased salt intake did not induce body fluid retention [35]. Rakova et al. [36] found higher salt intake reduced water intake, as water intake was ~300 mL/d higher during 6 g/d versus 12 g/d salt intake and the 6 g/d increase in salt did not increase urine volume. Bankir et al. [37] also reported urine volume remained unchanged over a large range of sodium intakes and that adaptation to a higher salt intake primarily increases urinary sodium concentration. As long as the sodium intake remains within 1150 to 5750 mg/day (the upper value, well above the recommended 2300 mg/d), urine flow rate does not appear to change [38]. Our participants’ mean sodium intakes were within the range of 2700 and 3500 mg/d. In short, except for the case of one male subject, the pre-test sodium intakes likely did not explain the poor reliability we observed in the diuretic action of repeated water ingestion. Moreover, the pre-test sodium intake likely did not impact our BHI repeatability results. The greater Na^+^ urine output observed on CAF was likely not due to the caffeine in the beverage per se, but appears consistent with the aforementioned studies that indicate when more Na^+^ is ingested (as in the test drink compared to plain water), urine [Na^+^] is higher resulting in greater Na^+^ excreted. However, we do not have the data from a non-caffeinated, sugar-free, electrolyte beverage trial to assess this directly.

From a general health standpoint, high dietary protein intake has been associated with an increased need for fluid intake. The only somewhat notable difference in our dietary intakes during water trial 2 vs. 1 was the higher mean dietary protein (27.5%). However, the mean relative protein intake was ~1.6 and 1.3 g/kg body mass on water trials 2 and 1, respectively. A controlled experimental trial that increased daily protein intake from 0.8 to 1.8 to 3.6 g/kg body mass for four weeks [39] did not impact indices of hydration status. In addition, in the aforementioned classical study, Luft et al. [34] found a daily protein intake of 80 compared to 180 g/day for 7 days (while holding caloric and sodium intake constant) did not affect water intake or urine output, further illustrating that neither sodium or protein fluctuations in a free-living diet in healthy adults correspond to meaningful changes in water intake or fluid balance. Therefore, any small deviation in protein intake was unlikely to impact our variable diuretic response to water and thus corresponding BHI results.

### 4.4. Seasonal Factors

Seasonal variations in changes in key hormones regulating fluid balance have been observed. Higher baseline antidiuretic hormone (ADH) and aldosterone during the summer months may occur as a compensatory mechanism to prevent dehydration from increased sweat loss and reduced electrolyte loss related to heat acclimatization [40]. Water 1 trial was initially conducted in late spring/summer months and water 2 trial during winter in the southeast United States; however, the directional responses in the diuresis from water trials were inconsistent based on season (6 of 15 were higher in summer). In our three outlier participants with the largest discrepancy in urine mass loss, only two had a higher diuretic action in winter, whereas the other individual had the opposite response (greater diuresis during summer). Whether seasonal variations fully explain the lack of repeatable urine mass loss reported here remains doubtful; however, it does imply that BHI trials in a single study with a large number of test beverages or conditions should ideally be conducted within a constrained time frame for each individual, i.e., not extending across major seasonal changes during participation in a BHI test protocol. If circumstances make this impractical, an extended period of time elapsing between trials may still be acceptable when executing a BHI protocol.

### 4.5. Caffeine Effects on BHI

The effect of caffeinated beverages, including energy drinks, was recently reviewed and cited as increasing urine production [31], all the while acknowledging that the data is of variable quality and demonstrates a need for future investigation. One study [41] reported greater urine volume (+243 mL) and urinary Na^+^ (23 mmol) after drinking 750 mL of RedBull^®^ energy drink (240 mg caffeine) vs. placebo over 6 h in 12 participants. Nussberger et al. [42] also found a 240 mg caffeine tablet increased diuresis and natriuresis. In both of these studies, caffeine was not paired with significant levels of electrolytes. The present study also found a short-term diuretic action for a sugar-free caffeinated electrolyte energy drink (60 min) that was below the water value of 1.0, but which did not persist in the typical BHI framework (over a timeline of 2–4 h). Hydration outcomes at 2–4 h for CAF were therefore similar to water, in alignment with other previous studies using the BHI protocol. In the original Maughan study [5], BHIs for caffeinated beverages (consisting of tea, coffee and diet cola) were also below 1.0, but not statistically different from water. In addition, increasing caffeine up to 400 mg in a beverage did not significantly impair BHI from 60 to 240 min [12]. Maughan and colleagues [12] also reported that increasing the carbohydrate content above 5% (to 10 and 20%) improved fluid retention of beverages using the BHI protocol. Whether the lack of sugar (or other macronutrients) amplifies any diuretic action of caffeine remains a possibility. Other energy drinks that had little to no sugar despite containing electrolytes also exhibited a lower BHI early in the post-ingestion observation period compared to a lower caffeine electrolyte product with a modest sugar (2–3%) content [11]. The sugar-free caffeinated electrolyte drink in this study also contained allulose, alanine, and glutamine. A previous study [10] found a sugar-free alanyl-glutamine dipeptide (2 g/L) electrolyte beverage (without caffeine) had a BHI (1.14) higher than water but only at 4 h. It should be noted that the BHI value previously deemed to be of practical importance as a threshold for differentiating a beverage’s hydration properties from water was 13% (i.e., a BHI < 0.87 or >1.13) in the literature [16]. No matter which water trial was used as the reference point, at no time did CAF exceed this threshold and only during the first hour was it below this BHI value (0.87). Sex differences in fluid retention with a caffeinated drink were not observed in this study, unlike our previous observation [11] which indicated women retained caffeinated beverages better than men. A similar magnitude of mean difference (~7% higher BHI ratio value for women) was observed for the BHI of CAF in water trial 2 and within the larger sample (*n* = 24), despite not finding a significant sex difference.

### 4.6. Limitations

This study was not specifically powered to test for sex differences and had unbalanced sample sizes for men and women. However, results tended to align with a previous study [10] suggesting a tendency for women to better retain a caffeinated beverage despite greater relative caffeine and fluid dose administered with a 1 L beverage. Since we failed to control the timing of BHI testing with the menstrual phase (or delimit to those only taking oral contraceptives) for better control of hormonal influences, no clear conclusion regarding potential female hormonal influences on the BHI of caffeinated beverages are possible. This study also did not systematically evaluate whether a trade-off in fluid retention exists between sugar/macronutrient content versus caffeine levels in a beverage based on the BHI test model. We were unable to control r these variables in the present study using a caffeinated beverage.

The use of the BHI model under the condition of euhydration has been criticized as a method of fully evaluating the hydration properties of a beverage, especially when rapid rehydration is desired. The real-world applicability of the BHI in this study is also limited, as it is unlikely that one liter of an energy drink would be exclusively consumed in a 30 min time period solely for hydration purposes. The relevance of BHI to clinically relevant situations arising from dehydration remains unclear. The BHI model fundamentally examines the end process of fluid ingestion due to fluid retention as a renal marker of hydration on a rather generalized time scale (hours compared to minutes when using blood biomarkers). Therefore, when time sensitivity is a lower priority under sedentary conditions, the BHI may be more appropriate.

## 5. Conclusions

Although mean urine output was not different in repeated water trials, the poor reproducibility for the diuretic action of water highlights individual variability, despite best efforts to control factors potentially influencing hydration status. However, there was no measurable impact on establishing the BHI of a sugar-free electrolyte beverage containing caffeine. Although the sugar-free caffeinated electrolyte beverage only elicited greater diuresis during the first hour, the BHI was no different than water thereafter, indicating comparable fluid retention at 2 and 4 h (the time periods considered to be the most relevant for hydration assessment). Therefore, these data suggest that a sugar-free, caffeinated electrolyte beverage supports long-lasting hydration despite containing caffeine. This may help address concerns around caffeine-associated dehydration while still delivering the desired functional benefits of caffeine. Moreover, the BHI for a sugar-free, caffeinated electrolyte beverage was not different between men and women. Therefore, the intra-individual variation in urine output observed during repeated water trials did not significantly impact the calculated BHI for a test beverage, indicating that the BHI metric is robust to interference from potential differences in individuals’ free-living conditions.

## Figures and Tables

**Table 1:** Mean (±SD) physical characteristics and habitual caffeine intake for *n* of 15 participants performing repeated water trials and the right column for the full sample of participants completing only one water trial versus CAF.

	Men (*n* = 10)	Women (*n* = 5)	Total (*n* = 15)	Total (*n* = 24)
Age	24.5 ± 4.0	22.4 ± 3.4	23.8 ± 3.8	22.9 ± 3.5
Height (cm)	181.9 ± 6.8	163.9 ± 8.4	175.9 ± 11.3	173.9 ± 10.7
Body Mass (kg)	76.5 ± 8.4	60.7 ± 8.3	71.2 ± 11.2	68.8 ± 10.7
% Body Fat	12.4 ± 5.2	22.3 ± 5.7	15.7 ± 7.1	16.2 ± 7.8
Total Body Water (L)	47.6 ± 5.9	33.1 ± 3.6	42.8 ± 8.7	41.5 ± 8.8
CAF (mg/kg/day)	1.1 ± 1.3	1.6 ± 0.7	1.3 ± 1.2	1.1 ± 1.1

**Table 2:** Mean (±SD) 24 h dietary records prior to each Beverage Hydration Index trial (*n* = 15). WAT = water. Nutrient intakes were not different (*p* > 0.05) across treatments.

Nutrient	WAT Trial 1	WAT Trial 2	CAF
Water (g)	3568 ± 1642	3709 ± 1647	3622 ± 1002
Energy (kcal)	2198 ± 850	2201 ± 627	2175 ± 764
Sodium (mg)	2734 ± 1336	2996 ± 1713	3422 ± 862
Protein (g)	91 ± 44	116 ± 48	100 ± 48
Carbohydrate (g)	279 ± 102	266 ± 94	261 ± 92

## Data Availability

Data are not publicly available but non-proprietary data may be made available upon request from the corresponding author.
